# Prediction of survival odds in COVID-19 by zinc, age and selenoprotein P as composite biomarker

**DOI:** 10.1016/j.redox.2020.101764

**Published:** 2020-10-20

**Authors:** Raban Arved Heller, Qian Sun, Julian Hackler, Julian Seelig, Linda Seibert, Asan Cherkezov, Waldemar B. Minich, Petra Seemann, Joachim Diegmann, Maximilian Pilz, Manuel Bachmann, Alireza Ranjbar, Arash Moghaddam, Lutz Schomburg

**Affiliations:** a Institute for Experimental Endocrinology, Charité-Universitätsmedizin Berlin, Corporate Member of Freie Universität Berlin, Humboldt-Universität zu Berlin, And Berlin Institute of Health, D-13353, Berlin, Germany; b HTRG, Heidelberg Trauma Research Group, Center for Orthopedics, Trauma Surgery and Spinal Cord Injury, Heidelberg University Hospital, D-69118, Heidelberg, Germany; c Department of General Practice and Health Services Research, University Hospital Heidelberg, D-69120, Heidelberg, Germany; d ATORG, Aschaffenburg Trauma and Orthopedic Research Group, Center for Orthopedics, Trauma Surgery and Sports Medicine, Hospital Aschaffenburg-Alzenau, D-63739, Aschaffenburg, Germany; e Institute of Medical Biometry and Informatics, Heidelberg University Hospital, Im Neuenheimer Feld 130.3, D-69120, Heidelberg, Germany; f Department of Allergy and Immunology, Mashhad University of Medical Sciences, Mashhad, Iran; g Institute of Interventional Allergology und Immunology, Bonn, Cologne, Germany

**Keywords:** Trace element, Inflammation, Micronutrient, COVID-19, Biomarker

## Abstract

SARS-CoV-2 infections cause the current coronavirus disease (COVID-19) pandemic and challenge the immune system with ongoing inflammation. Several redox-relevant micronutrients are known to contribute to an adequate immune response, including the essential trace elements zinc (Zn) and selenium (Se). In this study, we tested the hypothesis that COVID-19 patients are characterised by Zn deficiency and that Zn status provides prognostic information. Serum Zn was determined in serum samples (n = 171) collected consecutively from patients surviving COVID-19 (n = 29) or non-survivors (n = 6). Data from the European Prospective Investigation into Cancer and Nutrition (EPIC) study were used for comparison. Zn concentrations in patient samples were low as compared to healthy subjects (mean ± SD; 717.4 ± 246.2 vs 975.7 ± 294.0 μg/L, *P* < 0.0001). The majority of serum samples collected at different time points from the non-survivors (25/34, i.e., 73.5%) and almost half of the samples collected from the survivors (56/137, i.e., 40.9%) were below the threshold for Zn deficiency, i.e., below 638.7 μg/L (the 2.5th percentile in the EPIC cohort). In view that the Se status biomarker and Se transporter selenoprotein P (SELENOP) is also particularly low in COVID-19, we tested the prevalence of a combined deficit, i.e., serum Zn below 638.7 μg/L and serum SELENOP below 2.56 mg/L. This combined deficit was observed in 0.15% of samples in the EPIC cohort of healthy subjects, in 19.7% of the samples collected from the surviving COVID-19 patients and in 50.0% of samples from the non-survivors. Accordingly, the composite biomarker (SELENOP and Zn with age) proved as a reliable indicator of survival in COVID-19 by receiver operating characteristic (ROC) curve analysis, yielding an area under the curve (AUC) of 94.42%. We conclude that Zn and SELENOP status within the reference ranges indicate high survival odds in COVID-19, and assume that correcting a diagnostically proven deficit in Se and/or Zn by a personalised supplementation may support convalescence.

## 1 Introduction

A sufficient supply of essential micronutrients is of central importance for a fully functioning immune system, in part via enabling regular redox signalling by growth factors and cytokines [1]. However, the dietary intake of several trace elements and vitamins is often marginal, even in well-developed countries like the USA [2]. The situation is worse in many developing parts of the world with limited access to high-quality food and water, causing high rates of childhood deaths from common infections [3]. Given the current COVID-19 pandemic, it seems fortunate that young children are not particularly vulnerable to the Sars-CoV-2 virus, displaying generally unspecific symptoms only, and milder disease courses than adults [4]. Yet, the epidemiological experience with poorly nourished children has highlighted the importance and health-supporting potential of specific micronutrients in prevention and treatment of viral infections, e.g. positive effects of zinc (Zn) supplements in reducing acute lower respiratory tract infections [5], selenium (Se) supplementation in preventing virally-induced Keshan disease [6], or vitamin D in lowering acute respiratory tract infections [7]. Given these studies, it is speculated that adequate micronutrient status and supply may be of high importance in the current COVID-19 pandemic [[8], [9], [10]], and respectively designed supplementation studies have been suggested or initiated [[11], [12], [13]].

However, associations between micronutrient deficiencies and parameters of infection do not prove causality, as probably reflected best in the intensive and controversial discussion on the potential role of vitamin D deficiency in COVID-19 [[14], [15], [16], [17]], the possible perspectives and risks of vitamin D supplementation [[18], [19], [20], [21]], and respective misconceptions and misinformation [22,23]. While the current database from randomised controlled studies with vitamin D supplements in infectious and non-communicable diseases is abundant, other biologically active and potentially relevant micronutrients are less intensively studied [24]. This is an unfortunate situation, as some trace elements may be of particular relevance for the immune system, cytokine signalling and viral diseases like COVID-19, as reviewed recently in detail for the essential trace elements Zn [25,26] and Se [10,27]. At present, the actual concentrations, physiological roles and supplementation effects of these promising trace elements in COVID-19 are mainly discussed on a theoretical base, as clinical data are sparse.

One recent case report indicates that oral Zn supplementation would be tolerated well and may be associated with positive recovery from COVID-19 [28]. However, no laboratory parameters were assessed, controls were missing, and four patients only were reported. In a rural US. American setting, 93 of 150 (62%) COVID-19 patients received Zn supplementation along with other pharmacological treatments including or not hydroxychloroquine, azithromycin and/or plasma from convalescent subjects [29]. Again, the Zn status were not determined along with the supplementations, and no insights into any specific health effects of the Zn supplements could be deduced, as the interventions were not personalised, neither controlled nor blinded or monitored by laboratory parameters. Similarly, a retrospective analysis of 242 hospitalised COVID-19 patients receiving or not an average adjuvant dose of 100 mg Zn per day was neither reporting positive nor negative health effects, as the study was too small in size for the many confounders present, and Zn status of the patients was not determined or monitored [30].

A similarly poor database is currently available for the Se status and a potential positive health effect of Se supplementation in COVID-19. A seminal paper on the potential relevance of Se sufficiency in COVID-19 has indicated a strong linear relationship between cure rates and Se status of patients residing in geographically distinct areas of China with different baseline Se status [31]. This report is supported by a comparison of Se status in individual COVID-19 patients in relation to survival, highlighting that dynamic changes with time in the serum concentration of Se and its transporter selenoprotein P (SELENOP) provide relatively reliable information on mortality risk [32]. Both studies collectively indicate that Se deficiency may constitute a most relevant factor for COVID-19 course and prognosis. However, data from intervention trials with Se-containing supplements are missing. Nevertheless, the identification of a profound Se deficit in the majority of acute COVID-19 patients when arriving at the hospital indicates that the virus exerts substantial effects on the metabolism of Se by the host organism and that the deficiencies are unlikely a predisposition to virus infection but rather a consequence of disease [32]. In this study, we tested the hypothesis that Zn status is similarly affected by COVID-19, differs in relation to survival and may contribute to an improved prognosis.

## 2 Materials and methods

### 2.1 Study design

This cross-sectional study was conducted at two separate sites, including one public hospital involved in patient care and one research laboratory specialised in trace element analysis, i.e., the non-profit Public Hospital Klinikum Aschaffenburg-Alzenau, Germany, and the Institute for Experimental Endocrinology of Charité-Universitätsmedizin Berlin, Germany. Study details and diagnostic procedures have been published recently [32]. The study was conducted in accordance with the Declaration of Helsinki, and the authorities have provided ethical counselling in Bavaria, Germany (Ethik-Kommission der Bayerischen Landesärztekammer, EA No. #20033). The study was registered at the German Clinical Trial Register (Deutsches Register Klinischer Studien, ID: DRKS00022294), and written informed consent was provided by all patients enrolled or next of kin. On average, 4.9 ± 4.2 samples of blood were drawn consecutively per patient in the hospital and available for analysis by the research lab. Samples were kept at −80 °C or on dry ice during storage and transport. The laboratory analyses were conducted by scientists and technicians blinded to the patients' characteristics. In order to avoid bias, gain as much information as possible, and in view of the unpredictable number of patients, all samples available from the hospitalised cases of COVID-19 were analysed, without conducting a formal power analysis. Normal trace element ranges and reference values for serum concentrations of Zn, Se and SELENOP were derived from previous analyses conducted by the same technology of an extensive collection of serum samples from healthy adult subjects participating in the European Prospective Investigation into Cancer and Nutrition (EPIC) study [33,34].

### 2.2 Trace element analysis

Total reflection X-ray fluorescence (TXRF) analysis was used for quantitative Zn assessment in serum samples by a benchtop TXRF spectrometer (S4 T-STAR, Bruker Nano GmbH, Berlin, Germany) as described [[32], [33], [34]]. Briefly, samples were placed at room temperature to thaw, a gallium standard was added to each aliquot of serum, the solution was applied to polished quartz glass slides and dried, and a serum standard (Sero AS, Billingstad, Norway) served as a control in all assay runs. During the measurements, the intra- and inter-assay coefficients of variation (CV) were below 10%. The detailed analysis of serum Se and SELENOP status has been described recently [32].

### 2.3 Statistical analysis

Statistical analysis was performed with the open software R, version 4.0.2 [35], applying the packages “tidyr” [36], “dplyr” [37], “pROC” [38], “ggplot2” [39], and “caret” [40]. The Shapiro–Wilk test was used for assessing the normal distribution of values. Categorical variables were evaluated by Boschloo's test [41]. Comparisons of continuous variables were conducted by unpaired Student's t-test [42]. More than two groups were compared with ANOVA [43]. Correlations were tested by Spearman's correlation test [44]. Variable selection was performed via stepwise AIC selection [45,46]. Differences between ROC curves were assessed by the DeLong's test for two correlated ROC curves [47]. All statistical tests were two-sided, and *P*-values < 0.05 were considered significant; *p < 0.05, **p < 0.01, ***p < 0.001, and ****p < 0.0001.

## 3 Results

### 3.1 Patient characteristics

A total of n = 35 patients were enrolled in this observational study, providing a set of n = 171 consecutive serum samples, i.e., slightly enlarged as compared to our recent study [32]. COVID-19 patients who survived or died showed similar characteristics, except that surviving patients were on average considerably younger, as reported earlier and briefly shown here (Table 1).

**Table 1:** Characteristics of the COVID-19 patients contributing samples to this study.

	Death	Discharge	Total
Sex			
female	4 (67%)	15 (52%)	19 (54%)
male	2 (33%)	14 (48%)	16 (46%)
**Age**			
median (IQR)	89 (81, 94)	70 (38, 91)	77 (38, 94)
**Time to discharge or death**^a^**[d]**			
median (IQR)	10 (2, 32)	19 (3, 46)	15 (2, 46)

a Death in relation to COVID-19 diagnosis, irrespective of mortality cause; IQR, interquartile range.

### 3.2 Zinc (Zn) status analysis

**Fig. 1:**
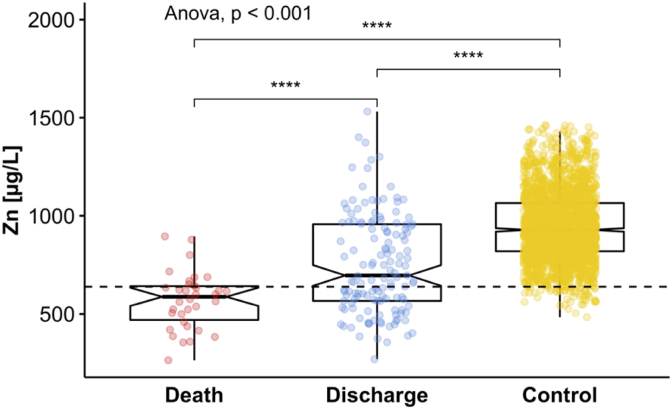
Comparison of Zn status in the available samples from patients with COVID-19 and healthy controls. Zn concentrations were relatively low in serum samples from COVID-19 patients in contrast to healthy European adults. Average Zn concentrations were particularly depressed in the samples from non-survivors as compared to the samples collected from patients surviving the SARS-CoV-2 infection. All available data are plotted into this figure, i.e., several data points refer to different samples from the same patients in the groups of deceased (“Death”) and surviving (“Discharge”) patients, respectively. The dashed line at <642.5 μg/L indicates the threshold for Zn deficiency. Groups of data were compared by ANOVA, **** indicates *p* < 0.0001.

Serum Zn status was evaluated from all COVID-19 samples and attributed to surviving patients or non-survivors. Results were compared to the EPIC study cohort, chosen to define the lab-specific reference range of serum Zn in healthy European adults. To this end, the data from EPIC were used to determine the interval encompassing 95% of the concentrations measured, defining the lower limit (Zn deficiency) at <642.5 μg/L represented by the bottom 2.5% of Zn values (Fig. 1).

Most of the samples from the COVID-19 patients were below the threshold for Zn deficiency. Notably, the samples from the non-survivors showed lower Zn levels than the samples from the survivors (Fig. 1), with some of the values indicating very severe Zn deficiency. Notably, some of the samples from the survivors showed an unsuppressed Zn status, above the median of the control EPIC cohort.

### 3.3 Zinc (Zn) deficiency in relation to biomarkers of Se status

**Fig. 2:**
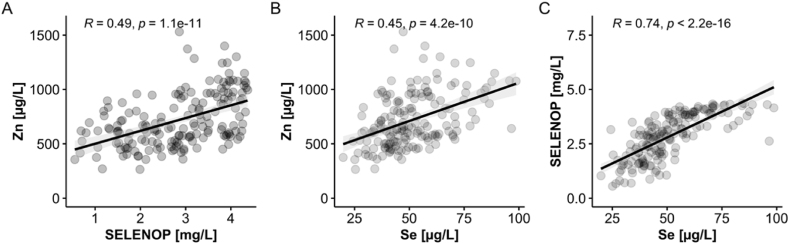
Correlation analyses of serum Zn concentrations with biomarkers of Se status in the samples from COVID-19 patients. All of the available serum samples (n = 171) from COVID-19 patients (n = 35) were analysed for total serum Zn and Se concentrations, as well as for serum SELENOP levels. (A) The concentrations of Zn and SELENOP showed a positive linear correlation (*R* = 0.49), in agreement with a comparable correlation between (B) total serum Zn and total serum Se concentrations (*R* = 0.45). The analysis of the data for (C) the Se transporter SELENOP and total serum Se concentrations showed the expected tight positive correlation (*R* = 0.74). *R*: Spearman correlation coefficient (2-sided, 2-tailed); *p* < 0.0001 for all three analyses (indicated on top of the graphics).

The extent of Zn deficiency in the samples analysed was reminiscent to the pattern observed before for serum Se and SELENOP concentrations, where both biomarkers of Se status were related to survival odds in COVID-19 [32]. To test for an interrelationship between Zn and the biomarkers of Se status, correlation analyses were conducted on all available data sets from the patient samples (Fig. 2). Surprisingly, not only the expected tight positive linear correlation between the two Se status biomarkers Se and SELENOP was observed (Fig. 2C), but also a positive correlation between serum Zn and serum SELENOP (Fig. 2A) as well as between serum Zn and serum Se (Fig. 2B) concentrations, respectively. This interrelationship in the samples from COVID-19 patients was far more pronounced than in the samples from the healthy subjects participating in the EPIC study, where the correlation coefficients were marginal (Zn/Se: *R* = 0.22; Zn/SELENOP: *R* = 0.06).

**Fig. 3:**
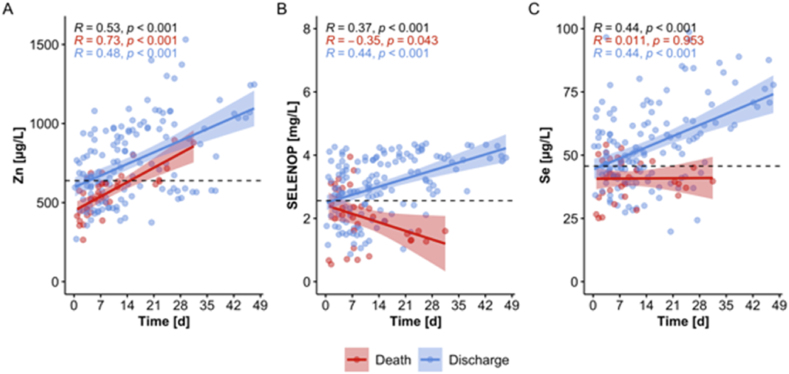
Dynamic changes in Zn levels in comparison to SELENOP and Se concentrations over the first weeks in hospital in the serum of COVID-19 patients in relation to survival. (A) Zn concentrations increased in the samples from COVID-19 patients irrespective of survival. (B) Serum SELENOP concentrations increased in survivors and decreased in non-survivors. (C) Serum Se concentrations increased with time in surviving patients only. Graphs B and C are slightly modified from the original publication and shown for comparison to Zn dynamics [32]. The dashed lines indicate the threshold of deficiency for the different biomarkers. Correlation analysis by Pearson, *R* indicates the correlation coefficient, and *p* indicates significance.

To identify potential parallel or discrepant kinetics between the parameters, alterations in serum Zn, Se and SELENOP concentrations were compared over the first weeks in the hospital in relation to survival (Fig. 3). The time-resolved patterns of the serum parameters showed some parallel positive development in the surviving patients but differed sharply in the COVID-19 patients who died. In contrast to the Se status biomarkers, Zn concentrations increased consistently in both survivors and non-survivors (Fig. 3A). Both biomarkers of Se status increased in the surviving patients (Fig. 3B and C), as reported earlier [32], but no increase was seen in the non-survivors. Notably, SELENOP concentrations decreased with time in the samples from non-surviving patients (Fig. 3B), whereas total serum Se concentrations remained relatively constant (Fig. 3C). These findings point to a potential relevance of time-dependent alterations of trace element biomarkers for predicting survival, particularly of dynamics in SELENOP concentration changes in COVID-19.

### 3.4 Simultaneous deficiency in Zn and Se or Zn and SELENOP in serum of patients with COVID-19

**Fig. 4:**
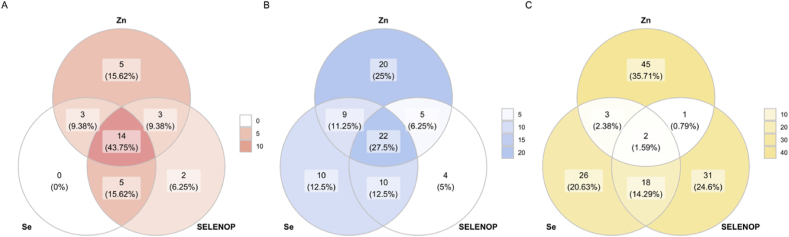
Analysis of combined deficits in the trace element parameters analysed in samples from COVID-19 patients in comparison to a reference cohort of samples from healthy subjects. (A) Patients not surviving the Sars-CoV-2 infection showed substantial deficits in the trace element biomarkers, with many of the analysed samples displaying relevant deficits in both Zn, Se and SELENOP. (B) In comparison, combined deficiencies in Zn, Se or SELENOP or combinations thereof were less prevalent in the samples from COVID-19 survivors. (C) In the large cohort of samples from healthy European subjects, simultaneous deficiencies in Zn and Se or Zn and SELENOP or all three biomarkers were very rare. In the diagram, the numbers of subjects deficient in at least one of the biomarkers are shown only, and the circles are not drawn to scale. The colours used are indicative of non-survivors (red), survivors (blue) or control subjects (yellow), with colour densities representing the prevalence of deficiency (according to the scale indicated to the right of each diagram). (For interpretation of the references to colour in this figure legend, the reader is referred to the Web version of this article.)

The analysis of Zn and Se status in serum of COVID-19 patients indicated that a profound acute decline in both parameters in response to the Sars-CoV-2 infection must have taken place. To compare and analyse the prevalence of a simultaneous deficit in Zn and SELENOP or Zn and Se in patients and controls, Venn diagrams were prepared (Fig. 4). In the samples from non-survivors, the prevalence of Zn deficiency was 73.5%, and a combined Zn and SELENOP or Zn and Se deficiency was detected in 50% of samples (Fig. 4A). Zn deficiency in surviving COVID-19 patients was found in 40.9% of samples, a combined Zn and Se deficit in 22.6% of samples, and a combined Zn and SELENOP deficit in 19.7% of samples (Fig. 4B). The prevalence of an isolated Zn, Se or SELENOP deficiency in the reference cohort of EPIC samples was per definition at 2.5% each. A combined Zn and Se or Zn and SELENOP deficit was a most rare event, present in less than 0.25% each in the full cohort of 2054 serum samples from healthy subjects (Fig. 4C).

### 3.5 Assessing the predictive value of serum Zn with biomarkers of Se status for predicting survival

**Fig. 5:**
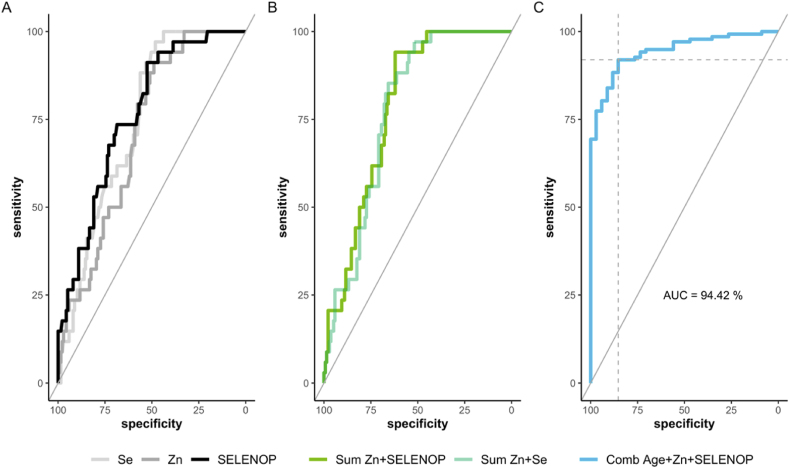
ROC-analysis of serum trace elements to predict survival of patients with COVID-19. (A) Serum concentrations of Se (pale grey), Zn (grey) and SELENOP (black) predict survival of patients with COVID-19 with similar and moderately good precision. (B) The sums of serum Zn with serum Se or serum Zn with serum SELENOP concentrations both yield a higher area under the curve (AUC). (C) The multiple regression model based on Zn, SELENOP and the patient's age outperformed any other combination of variables via stepwise AIC selection. The final model, based on these three parameters, yielded the highest AUC of 94.42%. The corresponding cutpoint, according to the Youden Index [48] is indicated.

Our analyses have indicated before that both biomarkers of Se status, i.e., total serum SELENOP and Se concentration, are related to survival odds in COVID-19 [32]. In the present study, their predictive value was compared in combination with serum Zn status and age. All three trace element biomarkers were tested separately and in different combinations by receiver operating characteristic (ROC) curve analysis to identify the most suitable isolated or compound prognostic parameter via stepwise AIC selection (Fig. 5). Then, the markers were combined by fitting logistic regression models. To avoid overfitting, the ROC curves were derived by five-fold cross-validation in the case where at least two parameters were combined. Hereby, a final logistic regression model was generated (Fig. 5C).

The ROC curve analyses indicate that the parameters serum Zn, serum Se and serum SELENOP concentrations were suitable to distinguish between those patients who could eventually be discharged and those who died, respectively, with AUC of 71.1% for Zn, 74.5% for Se, and 76.5% for SELENOP (Fig. 5A). Different combinations were tested and the resulting AUC for prediction of survival compared (Fig. 5B). By applying a stepwise Akaike information criteria (AIC) selection process, the combination of serum Zn and SELENOP concentrations with respect to the patients' age (Fig. 5C) outperformed the other variables as well as combinations thereof (Table 2).

**Table 2:** Specifics of the predictive models used.

	Model I: Se	Model II: Zn	Model III: Se + Zn	Model IV: SELENOP + Zn	Model V: Age, SELENOP, Zn
Intercept	1.76 ***	1.68 ***	1.84 ***	1.85 ***	6.40 ***
	[1.25, 2.26]	[1.20, 2.16]	[1.30, 2.37]	[1.31, 2.39]	[4.09, 8.72]
Se	1.21 ***				
	[0.62, 1.80]				
Zn		1.04 ***			0.93
		[0.49, 1.60]			[-0.04, 1.91]
Sum Zn + Se			1.34 ***		
			[0.74, 1.94]		
Sum Zn + SELENOP				1.33 ***	
				[0.77, 1.89]	
Age					−6.17 ***
					[-8.51, −3.83]
SELENOP					1.64 ***
					[0.78, 2.49]
N	171	171	171	171	171
AIC	151.78	155.89	146.18	143.46	82.49
BIC	158.07	162.17	152.46	149.74	95.05
Pseudo R^2^	0.20	0.16	0.24	0.26	0.68

All continuous predictors are mean-centered and scaled by 1 SD. ***p < 0.001.

## 4 Discussion

This study reports the Zn status of patients with COVID-19 and its relevance for predicting survival, as determined by the most common biomarker of Zn used in clinical studies, i.e., total serum Zn concentration. Our data indicate a profound and acute Zn deficiency in the majority of patients with COVID-19 when admitted to the hospital. The reference range used to define Zn deficiency in our analysis was derived from a large cross-sectional study of healthy European adults [34]. The thresholds are well in agreement with a Danish study where Zn was determined by flame-AAS (reference range determined by P2.5-P97.5; 536.1–1026.5 μg/L) [49], a study from Northern Ireland (reference range determined by P5–P95; 608.2–961.3 and 660.5–1111.8 μg/L, respectively) [50], and a recent study from Bangladesh (reference range determined by P2.5-P97.5; 600–1200 μg/L [51]). The analysis of the dynamic changes in serum Zn after hospital admission indicated an increase in Zn concentrations both in survivors and in non-survivors. This result was in contrast to our prior analysis of Se status biomarkers [32], where non-survivors displayed no improved levels in serum Se or SELENOP concentrations with time. In our former research, an increase in Se or SELENOP was specific for survival and a positive indicator of convalescence, much in agreement with analyses conducted before on the intensive care unit with patients suffering from SIRS or sepsis [52]. Importantly, our current analysis indicates that a combination of biomarkers yields substantial information on survival odds of COVID-19 patients, with the combination of serum Zn and serum SELENOP concentrations with patient's age outperforming other trace element-related parameters.

The consistent deficits observed for both serum Zn and Se in samples from newly admitted patients with COVID-19 point to an interfering and robust disrupting action of the virus on basic metabolic routes for these two essential trace elements. This finding may be related to the overlapping set of risk factors known to be associated with Zn and Se deficiency, respectively, i.e., senior age, malnutrition, hypoxaemia, comorbidities and/or chronic inflammation [26,[53], [54], [55], [56]]. Inversely, a deficit in Zn or Se is known to negatively affecting the immune system and adequate immune response to a challenge, potentially amplifying inflammatory signalling, losing the capacity for balanced responses and thereby harming the host tissue [27,57]. The mechanisms by which Zn is implicated in the immune system and potentially affecting the activity of immune cells are manifold, intertwined and beyond our current understanding, as best highlighted in several recent reviews summarising experimental evidence from cell culture or animal models and extrapolating the accumulated knowledge to the clinics and the current COVID-19 pandemic [25,26,[58], [59], [60]].

It is well established that micronutrients are of central importance for both the innate and the adaptive immune system [61]. Both Zn and Se support the differentiation, proliferation and normal function of T-cells. Furthermore, Zn promotes the phagocytic activity [62] and capacity [63,64] of monocytes, and supports the immunologic response and cytokine production of macrophages [65]. This notion is supported by recent evidence, suggesting that SARS-CoV-2 infections are associated with profound alterations in the myeloid compartment, with dysfunctional HLA-DRloCD163hi and HLA-DRloS100Ahi CD14^+^ monocytes prevailing in a severity-dependent manner in COVID-19 [66]. A redox-dependent Zn flux into myeloid cells, primarily monocytes and granulocytes, can be induced by lipopolysaccharide (LPS), phorbol-12-myristate-13-acetate (PMA) or other noxae [[67], [68], [69]]. Elevation of intracellular Zn levels, thereby affects immune cell activity by modulating a myriad of relevant Zn-dependent enzymes and transcription factors [57].

**Fig. 6:**
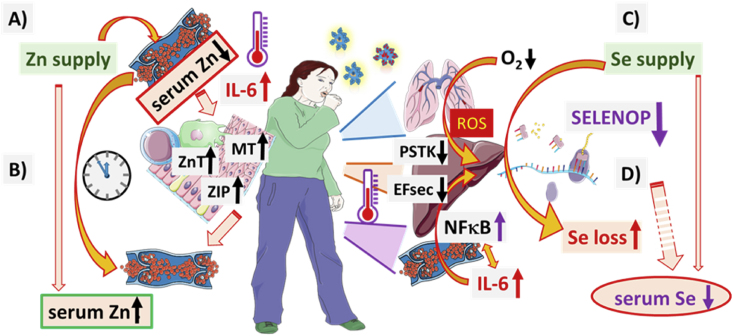
Overview of the physiological regulation and biochemical pathways potentially underlying the observed different dynamics of serum Zn and Se in COVID-19 in relation to survival or death.

Similarly, the molecular pathways affected by Se deficiency and potentially compromising the immune response to the infection by SARS-CoV-2 are numerous, and extrapolations from in vitro and animal experimentation predict a direct relevance of a sufficiently high Se status for successfully coping with COVID-19 [10,70,71]. Our present study now highlights that COVID-19 might be even more complicated than expected, as the majority of patients are displaying a simultaneous Zn and Se deficiency, i.e., a combined micronutrient deficit that is rarely addressed in preclinical experiments. Moreover, the kinetics indicate that the disease course is far more complicated than anticipated, with the apparent Zn deficit constituting an acute and reversible status with the deficit diminishing in response to medical care. In contrast, the Se deficit is resolving with time in surviving patients only. The profound difference in the dynamics of the two trace elements may be due to their dissimilar metabolism in response to a severe COVID-19 course, i.e., a reversible redistribution of Zn between serum and intracellular stores versus a reduced hepatic SELENOP release into blood due to impaired translation and down regulated biosynthesis (Fig. 6).

**A)** The regular nutritional supply of Zn maintains Zn status and enables the biosynthesis of Zn-dependent enzymes, transcription factors and structural proteins supporting the biochemical pathways of relevance for an appropriate antioxidative defence, energy metabolism and immune response. Increased inflammation and cytokine concentrations (in particular pro-inflammatory cytokines like IL-6) cause a fast and pronounced redistribution of Zn from serum into intracellular compartments by up-regulation of Zn-transporters (belonging to the ZnT and ZIP families) and intracellular Zn-binding proteins like metallothioneins (MT), particularly in immune cells and hepatocytes. **B)** After the initial strong decline in serum Zn status, a gradual release from the intracellular stores sets in, normalising serum Zn concentrations with time irrespective of ongoing COVID-19. **C)** The nutritional Se supply is first taken up by the liver, and a majority is inserted into SELENOP as an important and major transport protein for the trace element to extra-hepatic tissues. **D)** Under hypoxia and pro-inflammatory conditions, SELENOP biosynthesis is reduced, mainly on the translational level by decreased expression of rate-limiting factors including the O-phosphoseryl-tRNASec synthetase (PSTK) and the Se-specific elongation factor EFsec. Reduced SELENOP biosynthesis and diminished release from hepatocytes cause a loss of Se organification and a declining serum Se status. Recovery of SELENOP biosynthesis and protection from Se loss sets in with decreasing COVID-19 symptoms, whereas severe and deadly disease course is characterised by further Se loss and SELENOP decline. This figure was created by using some Servier Medical Art templates, which are licensed under a Creative Commons Attribution 3.0 Unported License; https://smart.servier.com.

The discrepant kinetics in Zn and Se status points to profoundly different molecular mechanisms responsible for the alterations detected. In several model systems, it has been observed that Zn becomes re-distributed within the organism in response to infection and severe inflammation. An up-regulation of hepatic Zn transporters in combination with elevated expression of intrahepatic metallothioneins as intracellular Zn-binding proteins contributes to an efficient translocation of serum Zn into the liver [24,26,[72], [73], [74]]. Besides, cytokine concentrations in inflammation, especially IL-6, are inversely related to serum Zn, partly also due to increased uptake into activated monocytes [75]. This redistribution out of serum into hepatocytes or monocytes is likely a reversible reaction. This notion would be compatible with the increasing levels of serum Zn observed after admission to the hospital and under improved medical care in both survivors and non-survivors. Diagnostic information on dynamic changes in serum Zn concentrations has also been described concerning neuronal damage [76,77]. However, in our analysis of samples from COVID-19 patients, the dynamics of changes in serum Zn concentrations were not related to survival. The substantial Zn deficiency observed already at the first time point after admission seems to provide the most valuable information about disease course and prognosis. Notably, a severe Zn deficit is generally known to associate with organ failure and mortality risk on the intensive care unit [78,79]. If the relation to organ failure also applies to COVID-19, then prophylactic or (adjuvant) therapeutic Zn supplementation should be considered as an acute, likely safe and well-tolerated measure. From the experiences in several intervention studies with critically ill patients, no side effects are expected in response to moderate dosage of supplemental Zn [59]. However, amounts of 200 mg/day should better not be surpassed over extended periods of time [80,81].

The molecular mechanism underlying the low Se status in COVID-19 is likely different. Dietary Se is mainly taken up by the liver and then distributed systemically in the form of SELENOP [82]. Under inflammatory conditions, in severe disease or under hypoxia, hepatic SELENOP biosynthesis is diminished, and Se metabolism is reduced [55,83,84]. As a result, a systemic Se deficit develops, as known from chronic hepatic diseases like hepatitis, cirrhosis or hepatocellular carcinoma [[85], [86], [87]]. This deficit seems not to constitute a reversible mechanism, as the organification of dietary or supplemental Se is impaired under inflammatory conditions, causing diminished uptake and metabolism of Se and increased loss of the precious trace element. A recovering Se status under disease conditions can instead be interpreted as a diminished inhibition of the impaired hepatic metabolism, causing an up-regulation of anabolic selenoprotein biosynthesis by hepatocytes and thereby an increasing SELENOP secretion and improved systemic Se transport. Lack of this up-regulation of hepatic selenoprotein biosynthesis indicates weak recovery and high mortality risk, as seen in COVID-19 and sepsis alike [32,52].

Given these considerations and the combined deficit observed at admittance to the hospital, a fast supplementation with a combination of Zn and Se should be considered as a promising adjuvant therapeutic measure, as both trace elements are severely low, especially in the non-survivors. However, clinical data on such supplementation in COVID-19 is not yet at hand, and a cautious approach to such an adjuvant treatment is therefore mandatory as discussed in more detail in recent review articles on this topic [26,88,89]. Conversely, an unsuppressed concentration of these two parameters of trace element status seems to indicate high survival chances. Ample experience with clinical supplementation of Se and Zn, respectively, is available, collectively indicating that such interventions can be considered as safe when adequate dosages are applied. However, the clinical experience with combinations of micronutrients in the clinics is scarce. There are rather reports of adjuvant supplementation of trace elements with other more controversial therapeutics like, e.g. (hydroxyl-)chloroquine, often mentioned in relation to Zn due to its effects on its distribution and uptake from serum [[90], [91], [92]]. Nevertheless, repurposing of established therapies appears currently as a promising option for COVID-19, and the correction of substantial deficits in immune-relevant micronutrients like Zn and Se may turn out as a most crucial parameter for survival. This notion may also apply to the long-term consequences of COVID-19, including issues like chronic fatigue as well as post-COVID cardiovascular and neurological problems like sensory impairment, where Zn and/or Se deficits are very likely relevant predisposing risk factors.

The particular strengths of the current study are the direct comparison of different trace element biomarkers by a standardised methodology in the same set of patients, and the blinded set-up of the analyses. Among the limitations are the relatively small group sizes and the lack of other relevant clinical parameters of the disease.

## 5 Conclusions

The combination of serum Zn and serum SELENOP concentrations with respect to the patient's age as one novel compound parameter of trace element metabolism provides valuable information on the prognosis of patients with COVID-19. Concentrations within the reference ranges indicate high chances for survival, whereas substantial deficiencies are reason to worry and to consider supplemental supply. Whether the correction of a strong trace element deficit in diseased patients supports convalescence in COVID-19 is currently unknown. This hypothesis is supported by the results of this study and should be tested in sufficiently sized, well-controlled and tightly monitored intervention studies in order not to miss a safe, cost-efficient and scientifically meaningful adjuvant treatment option.

## Author contributions

Conceptualisation, RAH, AM, and LS (Lutz Schomburg); methodology, RAH, QS, JS, JH, JS, LS (Linda Seibert), AC, WBM and PS; software, RAH, LS (Linda Seibert), AC, MP; visualisation, RAH, JS, LS (Linda Seibert) and MP; validation, RAH, QS, JH, JS and MP; formal analysis, RAH, PS, JD, MP, MB, AR, A.M., and LS (Lutz Schomburg); resources, MB, A.M. and LS (Lutz Schomburg); data curation, RAH, QS, JH, JS, MP, AM and LS (Lutz Schomburg); writing—original draft preparation, RAH and LS (Lutz Schomburg); writing—review and editing, QS, JH, JS, LS (Linda Seibert), AC, WBM, PS, JD, MP, MB, AR and AM; supervision, AM and LS (Lutz Schomburg); funding acquisition AM and LS (Lutz Schomburg). All authors have read and agreed to the published version of the manuscript.

## Funding

The research has been funded by the Deutsche Forschungsgemeinschaft (DFG), Research Unit FOR-2558 “TraceAge” (Scho 849/6-1) and CRC/TR 296 “Local control of TH action” (LocoTact, P17). We acknowledge funding received towards the doctoral thesis of RAH from the Oskar-Helene-Heim Foundation, Berlin, Germany.

## Declaration of competing interest

LS holds shares and PS serves as CEO of selenOmed GmbH, a company involved in Se status assessment and supplementation. The other authors declare no competing interest.
